# Capecitabine-Associated Coronary Vasospasm and Cardiac Arrest

**DOI:** 10.7759/cureus.28184

**Published:** 2022-08-19

**Authors:** Erind Muco, Haris Patail, Ayesha Shaik, Sean McMahon

**Affiliations:** 1 Internal Medicine, University of Connecticut School of Medicine, Farmington, USA; 2 Cardiology, Hartford Hospital, Hartford, USA

**Keywords:** ventricular fibrillation, breast cancer, coronary vasospasm, cardiac arrest, capecitabine

## Abstract

Capecitabine is a prodrug of fluorouracil that specifically targets cancer cells, commonly used as monotherapy for metastatic breast and colorectal cancer. Its side effects include nausea, diarrhea, vomiting, abdominal pain, anorexia, palmar-plantar erythrodysesthesia, anemia, and hyperbilirubinemia. Rarely, this chemotherapy agent has been associated with cardiotoxicity, including cardiac arrest, likely secondary to coronary vasospasm. This case report serves to highlight the unfortunate case of a 32-year-old female who suffered a ventricular fibrillation cardiac arrest three days after initiating capecitabine therapy.

## Introduction

Capecitabine is an important chemotherapeutic for colon and breast cancer. This case report discusses a 32-year-old female with a past medical history of estrogen receptor (ER)-negative, progesterone receptor (PR) weakly positive, and human epidermal growth factor receptor (HER)-negative invasive ductal carcinoma of the left breast. She was previously treated with neoadjuvant chemotherapy with carboplatin/paclitaxel, bilateral mastectomy, and radiation therapy. Since then, she began taking capecitabine three days before presenting to the emergency department with cardiac arrest. Several case reports highlight the relationship between capecitabine and cardiotoxicity and will be discussed further here. This case report serves to support the current theory that capecitabine-induced cardiotoxicity and subsequent cardiac arrest are due to coronary vasospasm.

## Case presentation

**Figure 1:**
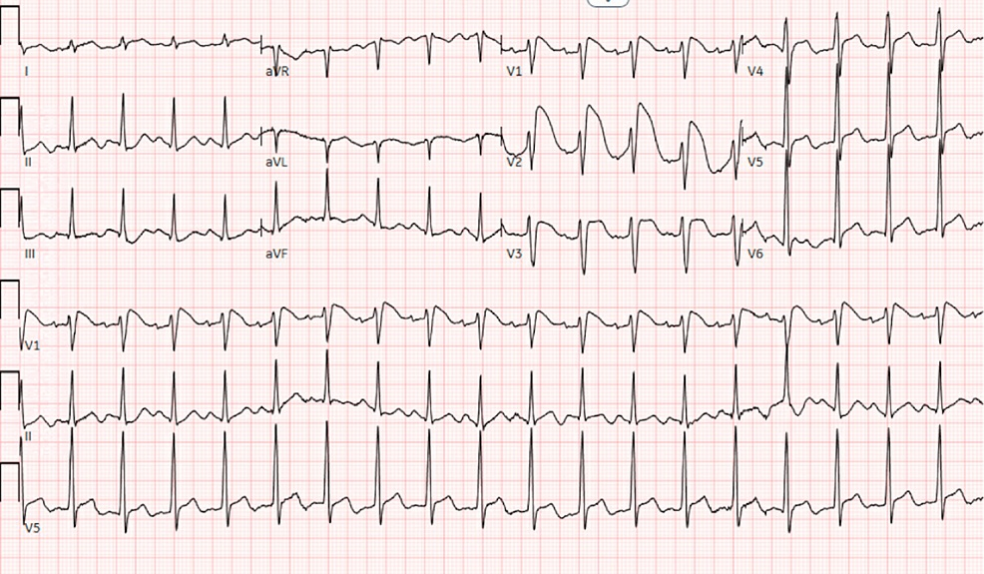
Initial EKG Upon presentation to the ED, the patient’s EKG showed concern for anterior ischemia.

**Figure 2:**
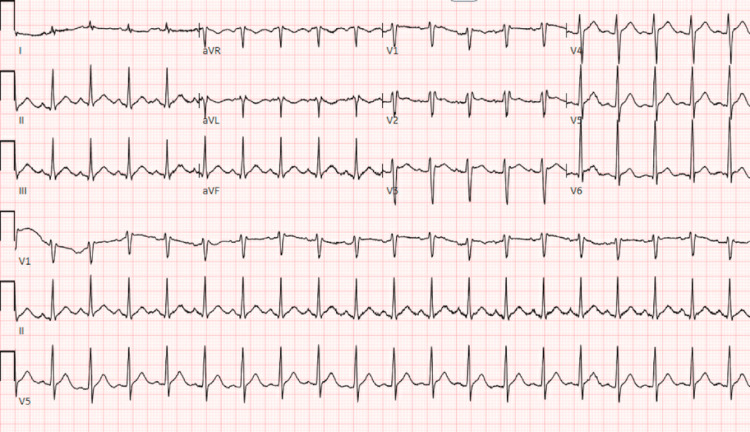
Subsequent EKG EKG obtained two hours after the initial one showed resolution of anterior ST elevations without any intervention.

The patient presented to the emergency department after collapsing at an outdoor event and was subsequently found to be in ventricular fibrillation (V-Fib) with alternating pulseless-electrical-activity (PEA) cardiac arrest. Bystander cardiopulmonary resuscitation (CPR) was initiated on the scene, and following the arrival of EMS, defibrillation and multiple rounds of epinephrine were given with successful return of spontaneous circulation (ROSC). The initial electrocardiogram (EKG) displayed in Figure 1 showed ST-segment elevations in leads I, V1-V6, and AVL, which were concerning for an anterolateral ST-elevation myocardial infarction (STEMI) versus Brugada syndrome. QTc was noted to be 504 milliseconds. The patient underwent emergent cardiac catheterization, which showed no significant coronary artery disease, dissection, or embolization of thrombus. CT angiogram of the chest was negative for pulmonary embolism. Initial laboratory work-up was unremarkable, including normal potassium of 4.0 mmol/L and magnesium of 2.9 mg/dL, the sensitive troponin assay peaked at 0.77 ng/mL and then down-trended. Repeat EKG shown in Figure 2 showed resolution of ST elevations, and transthoracic echocardiogram showed a left ventricular ejection fraction of 50-54%, enlarged right ventricle, and mid to apical anteroseptal hypokinesis. She underwent a targeted temperature management protocol in the cardiac intensive care unit. Her hospital course was complicated by nonconvulsive status epilepticus refractory to various sedatives (midazolam, propofol, and fentanyl) and anticonvulsants (levetiracetam and valproic acid). She had findings of focal anoxic brain injury noted on brain MRI and required gastrostomy and tracheostomy tubes for maintenance of her feeding and airway, respectively. She was eventually discharged to a long-term acute care facility with incomplete neurologic recovery at the time.

## Discussion

The most common adverse effects from capecitabine include lymphocytopenia (up to 94%), anemia (72-80%), palmar-plantar erythrodysesthesia (54-60%), diarrhea (47-57%), fatigue (≤42%), nausea (34-43%), and chest pain (≤6%). Cardiac arrhythmia, heart failure, cardiomyopathy, EKG changes, ischemic heart disease, and myocardial infarction have been reported, however, their frequency is not clearly defined.

We believe that the patient’s recent treatment with capecitabine chemotherapy precipitated her cardiac arrest. She had no pre-existing cardiac conditions, no evidence of cardiac ischemia on angiography, pulmonary embolism, electrolyte abnormalities, trauma, hypothermia, acidosis, infection, hypoxia, toxic ingestion, cardiac tamponade, or pneumothorax. Her initial EKG was concerning for ischemia due to ST elevations in the anterolateral leads, however, the Brugada pattern was also considered. Brugada became an unlikely differential diagnosis by first identifying that the patient had no family history of the disease or family history of sudden cardiac death. Furthermore, Brugada is rare in females and even rarer in younger females. Lastly, ST elevation in leads I, AVL, V5, and V6 are not typically seen in the Brugada pattern. A high precordial lead EKG was performed, which also did not show the Brugada pattern.

After ruling out other causes of cardiac arrest, as mentioned above, we then turned our focus to the patient’s history of breast cancer. The patient had undergone bilateral mastectomy and had been initiated on capecitabine three days before her cardiac arrest. It was noted from the patient’s family members and through accessing her oncology records that she was experiencing “burning substernal chest pain” within hours of starting capecitabine and this persisted up until her cardiac event. She was given metoclopramide and ondansetron by her oncologist without relief of symptoms.

As a prodrug of fluorouracil, capecitabine undergoes hydrolysis and is converted to the active form via the cytidine deaminase and thymidine phosphorylase enzymes, which are overexpressed in cancer cells. Therefore, the drug specifically targets cancer cells more than innate cells. Its labeled indications include monotherapy of metastatic breast cancer resistant to both paclitaxel and an anthracycline-containing regimen or resistant to paclitaxel in patients for whom further anthracycline therapy is not indicated [1]. There are reported cases of capecitabine-associated cardiotoxicity, including cardiac arrest [2-3]. In an American Heart Association (AHA) review on drugs associated with heart failure, Page et al. indicated that capecitabine is a known cardiotoxic drug [4]. Coronary vasospasm is the most well-established mechanism of cardiotoxicity in which there is vasoconstriction in vascular smooth muscle cells, however, some studies suggest that direct toxicity to the myocardium via capecitabine metabolites along with direct endothelial injury to the cardiac intima can lead to vascular thrombosis [5]. So far, there are limited data on the effect of capecitabine and fluorouracil chemotherapy agents on the cardiovascular system. In a small prospective study on patients without coronary artery disease treated with fluorouracil, 19 of 102 patients reported angina within 24 hours of initiating capecitabine therapy [5]. Furthermore, these patients had objective ST changes on EKG, which resolved within 24 hours of cessation of therapy. In another prospective clinical study, De Forni et al. observed the cardiotoxic effects of high-dose fluorouracil therapy in 367 patients where 7.6% of the patients developed cardiac events, which included (angina, arrhythmia, sudden cardiac death, and blood pressure variations) [6]. Of note, the median time for coronary vasospasm with fluorouracil is 12 hours post-infusion up to 72 hours [7-9].

## Conclusions

Our patient presented with cardiac arrest preceded by chest pain, dynamic ST changes, echocardiographic evidence of ischemia, and a negative coronary angiogram. Her cardiac event occurred within 72 hours of starting capecitabine, which supports a diagnosis of coronary vasospasm leading to ventricular fibrillation cardiac arrest from the chemotherapy agent. Of note, the median time for coronary vasospasm with fluorouracil is 12 hours post-infusion up to 72 hours. Treatment involves discontinuing the chemotherapy agent and using nitrates or calcium channel blockers if angina symptoms persist. Close monitoring and patient education regarding cardiac symptoms, such as chest pain, dyspnea, dizziness, and loss of consciousness post capecitabine or fluorouracil administration, are important. Further studies need to be conducted to better understand the dangerous cardiac effects of capecitabine given its common use for chemotherapy.
